# Study of the mass balance, biotransformation and safety of [^14^C]SHR8554, a novel μ-opioid receptor injection, in healthy Chinese subjects

**DOI:** 10.3389/fphar.2023.1231102

**Published:** 2023-09-14

**Authors:** Rupeng Shi, Yi Chai, Hao Feng, Lijun Xie, Lulu Zhang, Tianqi Zhong, Juan Chen, Peng Yan, Bei Zhu, Jun Zhao, Chen Zhou

**Affiliations:** ^1^ Phase I Clinical Trial Unit, The First Affiliated Hospital of Nanjing Medical University, Nanjing, China; ^2^ Value Pharmaceutical Services Co., Ltd., Nanjing, China; ^3^ Department of Clinical Pharmacology, School of Pharmacy, Nanjing Medical University, Nanjing, China; ^4^ Nuclear Medicine Department, The First Affiliated Hospital of Nanjing Medical University, Nanjing, China

**Keywords:** opioid receptor agonists, mass balance, metabolism, pharmacokinetics, radiolabel study

## Abstract

**Background:** SHR8554 is a novel μ-opioid receptor-biased agonist. It has analgesic effects by selectively activating the G protein-coupled pathway. Additionally, it can weakly activate the *ß*-arrestin-2 pathway, resulting in a limited number of side effects, such as gastrointestinal inhibition. Previous studies have shown that SHR8554 has good analgesic effects, safety and tolerability, but the pharmacokinetic characteristics of SHR8554 in humans have not been reported. This study was designed to investigate the pharmacokinetics and safety of SHR8554 in healthy Chinese male subjects.

**Methods:** A single 1 mg/41.3 μCi intravenous dose of [^14^C]SHR8554 was administered to six healthy male subjects. Blood, urine and faecal samples were collected at continuous time points to analyse SHR8554 parent drug levels and their metabolites. The total radioactivity in blood, plasma, urine and faeces was detected by using a liquid scintillation counter. The dynamic changes of SHR8554 and its metabolite concentration were by liquid chromatography-tandem mass spectrometry (LC/MS), and then pharmacokinetic analysis. The safety of the drug on the subjects was also observed after a single intravenous injection.

**Results:** The total recovery of radioactivity in urine and faeces was 99.68% ± 0.79% in 216 h, including 76.22% ± 1.12% in urine and 23.46% ± 1.36% in faeces. Seventeen major metabolites in blood, urine and faeces were analysed and identified. The main metabolic pathways of SHR8554 in the human body involve 1) N-dealkylation; 2) O-deethylation; 3) mono-oxidation; 4) glucuronidation, etc. The primary mechanism of SHR8554 clearance in the human body is through urinary excretion, primarily in its parent drug and metabolite forms. The drug has good safety, and no serious adverse effects were observed.

**Conclusion:** SHR8554 showed favourable pharmacokinetic characteristics and safety profiles in this study. SHR8554 is extensively metabolized in human body. The main metabolic pathways include N-dealkylation and O-deethylation, as well as mono-oxidation and glucuronidation. The main excretion route of SHR8554 and its metabolites is through urine.

**Clinical Trial Registration:** http://www.chinadrugtrials.org.cn/, identifier CTR20220450

## Introduction

SHR8554, a novel μ-opioid receptor biased agonist, is an innovative intravenous drug developed by Hengrui Pharma, Jiangsu, China. The main clinical indications were designed for moderate to severe acute pain.

Opioid agonists have been commonly used as analgesic drugs for moderate, severe, acute and chronic pain (Martínez-Navarro et al., 2019; Faouzi et al., 2020; Qiu et al., 2022). Traditional μ-opioid receptor agonists (e.g., morphine, dulcolax, fentanyl, etc.) are the more potent class of opioids in terms of their analgesic effects, which can activate both the G protein-coupled pathway and the *ß*-arrestin-2 pathway. Activation of the G protein-coupled pathway can produce powerful central analgesic effects, but activation of the *ß*-arrestin-2 pathway can cause adverse effects such as respiratory depression and constipation, which affects the clinical application of these drugs (Bohn et al., 1999; Raehal et al., 2005; Benyamin et al., 2008; Azzam et al., 2019).

Compared with conventional opioid receptor agonists, SHR8554 selectively activated the G protein-coupled pathway and weakly activated *ß*-arrestin-2 pathway (DeWire et al., 2013; Váradi et al., 2016; Schmid et al., 2017). These pharmacological characteristics enable SHR8554 to have good analgesic effects and fewer side effects.

Preclinical studies and preliminary clinical studies showed that SHR8554 had similar analgesic activity to morphine *in vivo* and *in vitro*, while the number of side effects of SHR855, such as gastrointestinal inhibition was significantly lower than that of morphine side effects. Moreover, SHR8554 has been shown to be safe and well tolerated in humans, and its effectiveness has been demonstrated in various degrees of postoperative pain, which suggests that SHR8554 has considerable clinical advantages as a biased μ-opioid receptor agonist.

To date, the metabolism, excretion and mass balance characteristics of SHR8554 in humans are not clear. The balance of radiolabeled substances in human body is the gold standard for studying the recovery, elimination and metabolic disposal of radiolabeled novel drugs after a single dosage (Penner et al., 2012; von Richter et al., 2016).

To further understand the mass balance and biotransformation pathways of SHR8554 in humans, we conducted a study in six healthy Chinese subjects to clarify the distribution, elimination and metabolism of SHR8554 after a single ^14^C-labelled intravenous dose.

## Methods

### Research design

The study was a single-centre, single-dose, open trial design in which six healthy Chinese male subjects were enrolled. Subjects received 1 mg/41.3 µCi [^14^C]SHR8554 injection on fasted state for 10 min in the morning of day 1 of the trial. Blood samples were collected 1 h prior to dosing and -24–0 h urine and faecal samples were collected; blood samples were planned to be collected from 0 to 144 h at specific time points. All excreted urine and faecal samples (the final collection time of samples was determined by the results of a staged assay) were collected from 0 to 216 h after dosing (Meng et al., 2019).

The aims of the study included detecting and analysing the pharmacokinetic (PK) parameters of [^14^C] SHR8554 and the parent drug SHR8554 as well as the ratio of total radioactivity in whole blood and plasma. The mass balance data were obtained, and the main excretion pathways in humans were identified by quantifying the total radioactivity in excreted samples from healthy subjects after the use of [^14^C] SHR8554. To identify the radioactive metabolite profile, the main metabolites in the plasma, urine and faeces of the subjects were detected after drug administration. The safety of [^14^C] SHR8554 in subjects after the use of a single dose of SHR8554 was also examined. This study was reviewed and approved by the Drug Clinical Trial Ethics Committee of the First Affiliated Hospital of Nanjing Medical University, and all subjects were fully informed and signed the informed consent form (ethical approval number: 2022-MD-093, drug clinical trial registration number: CTR20220450).

### Research methods

#### Participant selection

Six healthy male subjects were selected for this trial. The main inclusion criteria included the following: 1) healthy males between 18 and 45 years old; 2) a body mass index (BMI) value of 19.0–26.0 kg/m^2^; 3) regular bowel movements; 4) good health status, vital signs, physical examination, 12-lead ECG and laboratory tests were normal; 5) agreement to use highly effective contraception during the trial and for 12 months after drug administration. Major exclusion criteria included the following: 1) having cardiac, respiratory, gastrointestinal, endocrine, renal, skin, haematological, neurological or psychiatric diseases; 2) having a history of drug allergy; 3) having chronic constipation or diarrhoea, irritable bowel syndrome, inflammatory bowel disease, or perianal disease; 4) potential factors affecting drug absorption, distribution, metabolism and excretion; and 5) The subjects had previously participated in radiolabelled clinical trials or had recently been exposed to high levels of radiation (e.g., more than 1 session of X-ray, computed tomography scan, or barium meal, and being involved in radiation-related occupations) within the 12 months prior to the study.

#### Drug dose, preparation, and administration

According to the SHR8554 injection tolerability and PK findings from clinical phase I, phase II and III studies in healthy subjects, the nonradioactive SHR8554 administration dose was selected as 1 mg. Moreover, whole-body quantitative autoradiography (QWBA) data were obtained after injection of 0.6 mg/78 μCi/kg [^14^C] SHR8554 in male pigmented rats, and excretion data were obtained after administration of [^14^C] SHR8554 in male Sprague‒Dawley rats. The safe dose of [^14^C] SHR8554 for intravenous use in humans was estimated based on tissue distribution data from QWBA and excretion data from male Sprague‒Dawley rats after injection of 0.6 mg/78 μCi/kg [^14^C] SHR8554. The radiopharmaceutical dose of 41.3 μCi used in this trial falls within the dose range of the minor risk category of human radiation exposure. Furthermore, the estimated radiation dose values received by the lens, bone marrow, testes, uvea, and whole body of adult male subjects were much lower than the radiation dose limits defined by human Radiopharmacology studies (30 mSv) and specified by the United States Food and Drug Administration (US FDA) in 21 CFR 361.1. (Nijenhuis et al., 2016; Research, 2023; Title 21 of the CFR, 2023).

SHR8554 injection was produced by Jiangsu Hengrui Pharmaceutical Co., Ltd. (5 mL: 5 mg, stored below 30°C); [^14^C] SHR8554 injection was synthesized by Wuxi Beta Pharmaceutical Technology Co., Ltd. and isolated and purified by Jiangsu Wanliu Pharmaceutical Technology Co., Ltd. to prepare 0.316 mg/41.3 μCi [^14^C]SHR8554 injection, which was stored in a sealed refrigerator at 1°C–9°C. On the day of the experiment, the investigator diluted the SHR8554 and [^14^C] SHR8554 injection solution to achieve a 5 mL 0.9% sodium chloride injection at a dose of approximately 1 mg/41.3 μCi [^14^C] SHR8554 and administered it slowly intravenously for 10 min.

#### Sample collection and processing

Blood samples: The endpoint of blood sample analysis was drug radioactivity concentration <3 times the plasma background value in blood samples at two consecutive time points. Venous blood samples were collected from 6 subjects at 0 h (within 1 h before dosing) and immediately after intravenous infusion, 15 min, 30 min, 1 h, 1.5 h, 2 h, 3 h, 4 h, 6 h, 8 h, 12 h, 24 h, 96 h, and 144 h. Plasma samples were obtained after centrifugation (2°C–8°C, 1,500 g ± 10 g for 10 min). All plasma and whole blood samples were stored frozen in a refrigerator (−10°C ∼ −30°C) for further testing (Liao et al., 2020).

Urine and faecal samples: In this experiment, urine and faecal samples were collected as follows: 1) Urine Samples Collection: collect single urine samples from the subjects during the pre-dosing period (−24 to 0 h), and collect all excreted urine within different time intervals after dosing (0–4 h, 4–8 h, 8–12 h, 12–24 h, 24–48 h, 48–72 h, 72–96 h, 96–120 h, 120–144 h, 144–168 h, 168–192 h, 192–216 h). 2) Faecal Samples Collection: collect single faecal samples from the subjects during the pre-dosing period (−24 to 0 h), and collect all excreted faecal samples within different time intervals after dosing (0–24 h, 24–48 h, 48–72 h, 72–96 h, 96–120 h, 120–144 h, 144–168 h, 168–192 h, 192–216 h). Urine and faecal samples were collected at the endpoint, which was determined by a total excretion of radioactivity exceeding 80% of the administered dose and a cumulative excretion of the administered dose on two consecutive days of <1%. The urine and faecal samples were collected from subjects 1,001–1,004 before and 0 h–216 h after dosing and from subjects 1,005–1,006 before and 0 h–168 h after dosing; subject 1,003 vomited after dosing, and the vomitus was collected from 0 h to 8 h. The collected specimens were weighed in special containers and temporarily stored in a refrigerator at −20°C before measurement (Bian et al., 2021).

#### Analytical measurements

##### Main instruments

TRI-CARB 4910 TR liquid scintillation counter (Perkin Elmer, Wellesley, MA)were used for measuring radioactivity. OX-501 oxidizer (R.J. Harvey, Tappan, NY), 20 HPLC system or 30-AD XR HPLC system (Shimadzu Co., Kyoto, Japan), Packard TopCount ^®^ NXT™ Microplate Scintillation and Luminescence Counter and v. ARC™ Online Radioisotope Detector (Model2, AIM Research Corporation, USA) also used in the present study.

##### Radioactivity of samples

The radioactivity of plasma, whole blood, urine and faecal samples was quantified using a liquid scintillation counter (LSC). In this study, plasma and urine samples were obtained and mixed with a certain amount of scintillation solution, and their radioactivity was verified by an LSC; whole blood and faecal homogenate samples were fully combusted by an oxidative combustion apparatus, and the [^14^C] carbon dioxide generated by the combustion of the samples was captured with a special scintillation solution for [^14^C], and then the amount of radioactivity contained was determined by an LSC. Vomitus was subjected to extraction by the addition of 1–2 times the volume of organic solvent to obtain the supernatant, and the amount of radioactivity in the sample was measured using LSC. The total radioactivity in urine and faeces was measured using an LSC, and the cumulative rate of drug excretion was calculated based on the weight of urine and faeces collected periodically and the concentration of radioactive material. PK analysis was performed, and the total radioactivity ratio of whole blood to plasma was calculated based on the total radioactivity intensity in plasma and whole blood. During the sample preparation process, we calculated the radioactive extraction efficiency and recovery rate.

##### PK assay

The blood concentration of the SHR8554 parent drug in plasma was detected and quantified using a LC/MS method, and PK analysis was performed.

##### Metabolite detection and analysis

The samples of plasma, urine, and faecal collected at different time intervals are mixed using specific methods. After obtaining the mixed samples, they are processed appropriately for radioactive metabolism spectrum research. Plasma samples were collected from each subject at pre-dose and post-dose time points of 0.167 h, 0.25 h, 0.5 h, 1 h, 1.5 h, 2 h, 3 h, 4 h, 6 h, 8 h, 12 h, 24 h, and 48 h. Using the AUC method for each time point, a portion of the plasma sample was taken and mixed to obtain six mixed plasma samples covering the time range from 0 h to 48 h. Urine samples from six subjects are mixed using the same volume percentages to create six mixed urine samples covering the time range from 0 h to 96 h. Based on time intervals, three urine samples are obtained by mixing with the same volume percentages, covering 0 h–12 h, 12 h–48 h, and 48 h–96 h. Faecal samples from six subjects are mixed using the same weight percentages to create six mixed faecal homogenate samples covering the time range from 0 h to 120 h. Based on time intervals, three faecal homogenate samples are obtained by mixing with the same weight percentages, covering 0 h–48 h, 48 h–72 h, and 72 h–120 h. These processed samples will be used for radioactive metabolism spectrum research.

Plasma, urine and faecal homogenates were extracted and concentrated by organic solvents, and then the redissolved solutions were utilized for metabolite profile analysis. The plasma, urine and faecal extracts were separated by HPLC columns, and the HPLC flow fractions were collected in Deepwell LumaPlateTM-96 plates at 15 s/fraction using an automatic fraction collector. The radioactivity of each fraction was quantified using a microplate detector with ARC®Convert and Evaluation software (version no. ARC3 Ver 3.0.2.379) and transformed to obtain the radioactive metabolite spectra. The radioactive metabolite spectra of each sample were integrated to acquire the peak area of each radioactive metabolite peak, determine the proportion of each spectral peak to the total radioactive intensity of the sample (%HPLC), and obtain the proportion of the radioactivity of the major metabolites to the total radioactivity in the corresponding blood, urine and faecal samples by calculation. Therefore, the ratio of circulating concentrations of metabolites in plasma to total exposure to AUC and the ratio of levels of each metabolite in urine and faecal to the dose administered were calculated. Liquid chromatography with low-energy radionuclide detection and mass spectrometry (LC/RAM/MS) were used to identify the main metabolites in human plasma, urine and faecal samples and to identify the possible biotransformation pathways of SHR8554 in humans.

#### Safety evaluation

Adverse events were defined according to the U.S. Department of Health and Human Services Criteria for the Evaluation of Commonly Occurring Adverse Events (CTCAE) version 5.0 (Adverse Events/CTCAE | CTEP, 2023), and indicators included drug-related adverse reactions (ADRs), laboratory test results (including from blood routine, urine, faecal, blood biochemistry and coagulation function), vital signs (temperature, pulse, respiratory rate, arterial blood pressure, blood oxygen saturation), physical examination results, 12-lead electrocardiogram, and other indicators.

## Statistical analysis

Microsoft Office Excel (version 2010) was used to calculate data on the proportion, mean and standard deviation of radioactivity in urine and faecal samples in relation to the actual dosing, and to plot the cumulative excretion ratio curves of total radioactivity in urine and faeces; Phoenix WinNonlin^®^ software (version 8.3, Pharsight) was applied to calculate the total radioactivity in whole blood and plasma and the PK parameters of SHR8554 according to the noncompartment model, including area under the drug-time curve (AUC_0-t_ and AUC_0-∞_), time to peak (T_max_), peak concentration (C_max_), elimination half-life (t_1/2_), elimination rate constant (*λ*_z_), mean retention time (MRT), apparent clearance (CL), and final apparent distribution volume (V_z_). SAS 9.4 software was used to analyse the data, and the data are presented as the mean ± standard deviation (X ± SD).

## Results

### Demographic and safety results

All 6 male subjects were Han Chinese, aged 23–30 years and a mean age of 27.5 ± 2.43 years and had a body mass index (BMI) ranging from 20.3 to 25.6 kg/m^2^ with a mean of 23.42 ± 1.95 kg/m^2^. Six subjects completed this clinical trial, and 4 cases of adverse events (16.7%) occurred in 1 subject (1003), all of which were treatment-related adverse events (TRAEs). The incidence of TRAEs was 16.7% (1/6) and included dizziness, vomiting, nausea, and weakness. Of these, vomiting was a moderate TRAE and was treated with the drug 5% dextrose sodium chloride, while the remaining TRAEs were mild, and recovery from all TRAEs occurred. None of subjects experienced serious adverse events, and no subjects withdrew from the study related to adverse events.

### Actual dose administered

The total amount of radioactivity actually used per subject was acquired by multiplying the amount of radioactivity in each Gram of preparation by the weighed sample size of each preparation and subtracting the amount of radioactivity remaining in the container after administration. In turn, the actual dose administered was calculated based on the specific activity. The intended dose administered was approximately 1 mg/41.3 µCi, and six subjects actually received 0.911 mg–0.957 mg of SHR8554 with a mean value of 0.941 mg ± 0.0178 mg and a radioactive dose of 39.4 µCi–41.4 µCi with a mean value of 40.7 µCi ±0.771 µCi.

### PK results

#### Total radioactivity (TRA) in whole blood and plasma and blood concentrations of the SHR8554 parent drug in plasma

**FIGURE 1:**
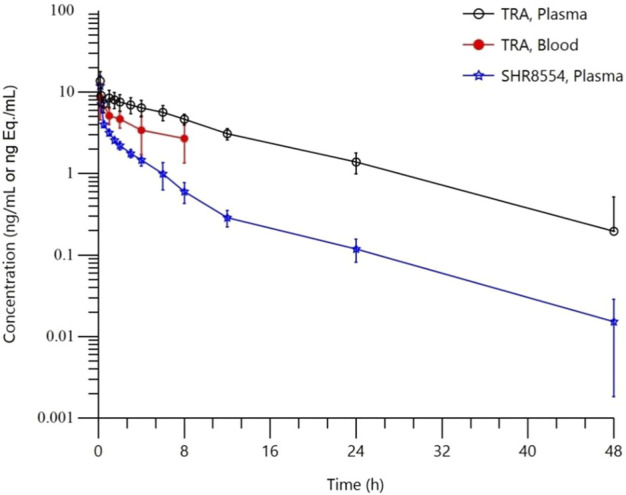
Mean blood concentration-time semilog curves. Note:Data are represented as the mean ± standard deviation in whole blood, total plasma radioactivity and plasma SHR8554 concentration after a single dose of 1 mg/41.3 μCi [14C]SHR8554 (n = 6 subjects).

The mean blood concentration-time semilog curves for total radioactivity (TRA) in whole blood and plasma and for the SHR8554 parent drug are shown in Figure 1.

#### TRA in plasma and the main PK parameters of the SHR8554 parent drug

The TRA in plasma and the main PK parameters of the SHR8554 parent drug following a single intravenous administration of [^14^C] SHR8554 in healthy Chinese male subjects are shown in Table 1. The median peak time (Tmax) of SHR8554 in human plasma was 0.167 h, and the mean peak concentration (Cmax) was 11.9 ± 3.81 ng/g (mean ± standard deviation). After reaching the peak, the concentration of SHR8554 gradually decreases, and after 48 h of administration, the plasma concentrations of SHR8554 in two subjects were below the quantification limit (0.0143 ng/g). The mean area under the plasma concentration-time curve (AUC0-t) of SHR8554 is 20.8 ± 2.29 h•ng/g, the mean residence time (MRT0-t) was 6.02 ± 1.30 h, the estimated elimination half-life (t1/2) was 8.24 ± 2.83 h, the apparent volume of distribution at steady state (Vz) of SHR8554 was 564 ± 199 L, and the apparent clearance rate (CL) is 47.6 ± 5.45 L/h.

**TABLE 1:** Summary of PK parameters of total radioactivity and SHR8554 parent drug levels in plasma (n = 6 subjects).

Parameter	Unit	Total plasma radioactivity	SHR8554
T_max_ ^a^	h	0.167 (0.167,1.00)	0.167 (0.167,0.167)
C_max_	ng Eq./g^b^	13.8 ± 3.94	11.9 ± 3.81
AUC_0-t_	h·ng Eq./g^b^	104 ± 26.9	20.8 ± 2.29
AUC_0-∞_	h·ng Eq./g^b^	118 ± 28.8	21.2 ± 2.30
MRT_0-t_	h	9.49 ± 2.71	6.02 ± 1.30
t_1/2_	h	10.4 ± 3.11	8.24 ± 2.83
λ_z_	1/h	0.0709 ± 0.0169	0.0937 ± 0.0346
V_z_	L	127 ± 22.5	564 ± 199
CL	L/h	8.85 ± 1.96	47.6 ± 5.45

^a^ Median values (min and max) are reported in the table.

^b^ C_max_ and AUC, for parent drug and metabolites are in ng/g and h-ng/g, respectively.

The median peak time (Tmax) for total plasma radioactivity (equimolar to SHR8554) was 0.167 h, which is the same as that of the parent drug. The average peak concentration (Cmax) was 13.8 ± 3.94 ng Eq./g (mean ± standard deviation). After reaching the peak, the radioactive concentration gradually decreases, and only a small amount of radioactivity can be detected in the plasma of two subjects after 48 h of administration. The plasma of the remaining four subjects was below the quantification limit (0.438 ng Eq./g). The average area under the curve (AUC0-t) for total plasma radioactivity was 104 ± 26.9 h ng Eq./g, the mean residence time (MRT0-t) is 9.49 ± 2.71 h, the estimated average half-life (t1/2) was 10.4 ± 3.11 h, the average apparent volume of distribution (Vz) was 127 ± 22.5 L, and the average clearance rate (CL) was 8.85 ± 1.96 L/h.

#### Total radioactivity ratio of whole blood to plasma

Only a small amount of radioactivity was detectable in the plasma of two subjects 48 h after administration, and the levels in the remaining four subjects were below the lower limit of quantification (0.438 ng Eq./g). The total radioactivity ratio of whole blood to plasma for the quantifiable samples was less than 1 and ranged from 0.370 to 0.813, as shown in Table 2, suggesting that [^14^C] SHR8554-related substances did not bind considerably to blood cells.

**TABLE 2:** Ratio of whole blood to total plasma radioactivity after dosing in six healthy Chinese male subjects.

Time h)	Total radioactivity ratio of whole blood to plasma							
	1001	1002	1003	1004	1005	1006	Mean	Standard deviation
0	NA	NA	NA	NA	NA	NA	NA	NA
0.167	0.370	0.747	0.628	0.813	0.596	0.538	0.615	0.157
1	0.519	0.556	0.595	0.636	0.667	0.677	0.608	0.0630
2	0.465	0.613	0.579	0.727	0.645	0.697	0.621	0.0935
4	NA	0.590	0.516	0.676	0.686	0.720	0.638	0.0832
8	NA	0.662	0.602	0.702	0.700	0.768	0.687	0.0608

Note: NA, not applicable.

### Mass balance and excretion results

**FIGURE 2:**
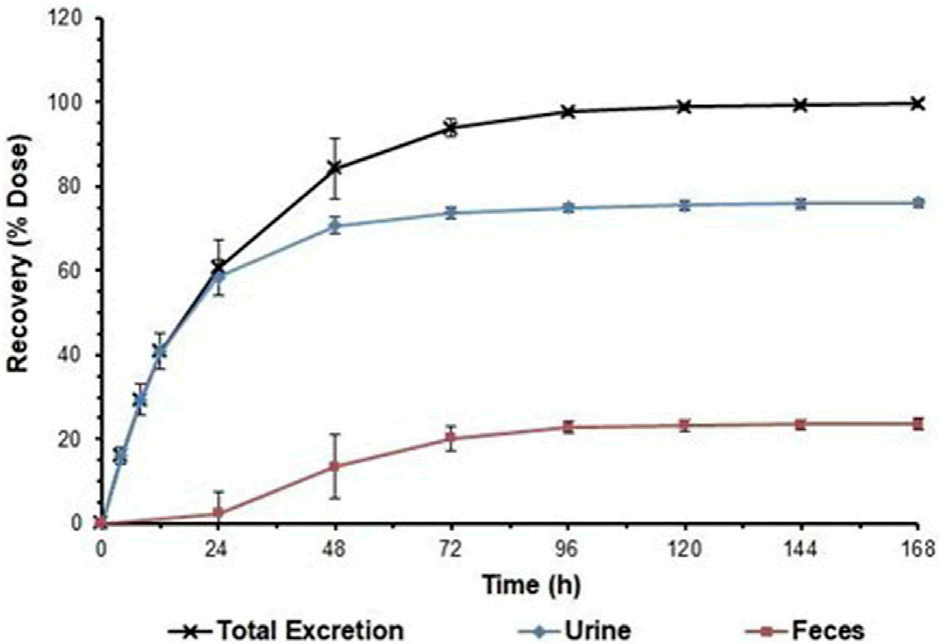
Cumulative excretion ratios in urine and faeces. Note:Data are represented as the mean ± standard deviation after a single dose of 1 mg/41.3 μCi [14C]SHR8554. (n = 6 subjects).

The total radioactivity recovered from urine and faeces after administration accounted for 99.68% ± 0.79% of the dose administered. Total radioactivity was detected mainly in urine (76.22% ± 1.12% of the administered dose), and partially in faeces (23.46% ± 1.36%). In one of the subjects (1,003), vomiting occurred between 0 and 8 h after administration, and the total amount of radioactivity recovered in the vomitus was 1.92% of the dose administered. As demonstrated in Figure 2, excretion of total radioactivity in urine and faeces occurred mainly within 72 h (3 days) after dosing, with the average excretion during this period accounting for approximately 93.90% of the dose administered, and the daily excretion rates after 120 h of dosing were all less than 1% of the dose administered.

### PK characteristics of SHR8554 in humans

#### Metabolites of SHR8554 in humans

In healthy male subjects, after a single intravenous injection of 1 mg/41.3 μCi [^14^C]SHR8554 injection solution, urine was the major clearance pathway. The total radioactivity excreted in urine from 0 h to 216 h accounted for 76.22% of the administered dose, with the identified radioactivity peaks in urine accounting for 69.42% of the administered dose (or 91.08% of the urine). The parent drug accounted for only 3.69% of the administered dose in urine. The main metabolites in urine were M2, M14-1, and M14-2, accounting for 14.92%, 8.13%, and 21.20% of the administered dose, respectively. The minor metabolites were M6-1, M6-2, M12, M13, M18-1, M18-2, M18-3, and M20, accounting for 1.62%, 1.05%, 2.99%, 1.47%, 3.82%, 3.67%, 3.40%, and 2.59% of the administered dose, respectively. The identified other metabolites M7-1, M7-2, and M7-4 accounted for less than 1% of the administered dose in urine. The unidentified radioactive areas were not higher than 3% of the administered dose.

After a single intravenous injection of 1 mg/41.3 μCi [^14^C]SHR8554 injection solution, partial excretion was via faeces. The total radioactivity excreted in faecal sample from 0 h to 216 h accounted for 23.46% of the administered dose, with the identified radioactivity peaks in faeces accounting for 11.96% of the administered dose (or 50.98% of the faeces). The parent drug accounted for only 0.12% of the administered dose in faeces. The major identified metabolites in faeces were M7-2, M7-3, and M14-2, accounting for 3.41%, 2.37%, and 2.67% of the administered dose, respectively. Six other identified metabolites in faeces, namely, M2, M4, M6-2, M7-1, M7-4, and M12, were all less than 1% of the administered dose. The unidentified radioactive areas were not higher than 1% of the administered dose.

The parent drug is the major radioactive component in plasma, accounting for 35.29% of the total radioactivity exposure in plasma. The main circulating metabolites in plasma were M6-2, M7-2, and M13, which accounted for 10.50%, 9.65%, and 11.69% of the total radioactivity exposure, respectively. The minor metabolites were M2, M3-2, M4, M6-1, M7-4, M12, M18-2, and M18-3, accounting for 1.42%, 3.72%, 1.41%, 2.93%, 1.63%, 4.37%, 1.12%, and 5.42% of the total radioactivity exposure in plasma, respectively. The other trace metabolites M7-1 and M14-2 were both less than 1% of the total radioactivity exposure in plasma. The unidentified radioactive areas were not higher than 5% of the total radioactivity exposure in plasma.

The results for the levels of the parent drug and its metabolites as a percentage of total plasma radioactivity exposure (% AUC) and in urine and faeces as a percentage of dose administered (% Dose) in healthy male subjects are shown in Table 3.

**TABLE 3:** Summary of the levels of [^14^C] SHR8554 and its metabolites in total plasma radioactive exposure (% AUC) and in urine and faeces as a percentage of dose (% dose).

	Metabolite type	Plasma (0–48 h)		Urine (0–216 h)	Faeces (0–216 h)	Faeces + urine
		%AUC	Metabolite/Parent drug (%)^a^	%Dose (76.22%)	%Dose (23.46% *^b^*)	%Dose (99.68% *^b^*)
SHR8554	Parent drug	35.29	100	3.69	0.12	3.81
M2	N-dealkylation	1.42	4.02	14.92	0.88	15.80
M3-2	O-deethyl and dehydrogenated	3.72	10.54	ND	ND	ND
M4	O-deethylation	1.41	4.00	ND	0.03	0.03
M6-1	M3 mono-oxidation	2.93	8.30	1.62	ND	1.62
M6-2	M3 mono-oxidation	10.50	29.75	1.05	0.78	1.83
M7-1	M4 mono-oxidation	0.88	2.49	0.35	0.29	0.64
M7-2	M4 mono-oxidation	9.65	27.34	0.36	3.41	3.77
M7-3	M4 mono-oxidation	ND	NA	ND	2.37	2.37
M7-4	M4 mono-oxidation	1.63	4.62	0.16	0.68	0.84
M12	mono-oxidation	4.37	12.38	2.99	0.73	3.72
M13	M12 glucuronidation	11.69	33.13	1.47	ND	1.47
M14-1	M2 mono-oxidation	ND	NA	8.13	ND	8.13
M14-2	M2 mono-oxidation	0.61	1.73	21.20	2.67	23.87
M18-1	M7 glucuronidation	ND	NA	3.82	ND	3.82
M18-2	M7 glucuronidation	1.12	3.17	3.67	ND	3.67
M18-3	M7 glucuronidation	5.42	15.36	3.40	ND	3.40
M20	N-dealkylation and carboxylation	ND	NA	2.59	ND	2.59
Total identification peaks^c^		90.64		69.42	11.96	81.38
Total unidentified peaks^d^		9.36		6.80	11.50	18.30
Percentage of peaks identified^e^		90.64		91.08	50.98	81.64

Note.

^a^ The percentage of metabolites relative to the parent drug in plasma.

^b^ The percentage of urine and faeces excretion from 0 to 216 h relative to the administered dose.

^c^ The percentage of identified peaks in plasma relative to the total radioactivity exposure or dose administered.

^d^ Individual radioactive peaks do not exceed 5% of the AUC, or 3.00% of the dose.

^e^ The sum of identified peaks as a percentage of the current matrix.

NA, not applicable.

ND, not detected.

#### Quantitative metabolite profiling of SHR8554 in humans

**FIGURE 3:**
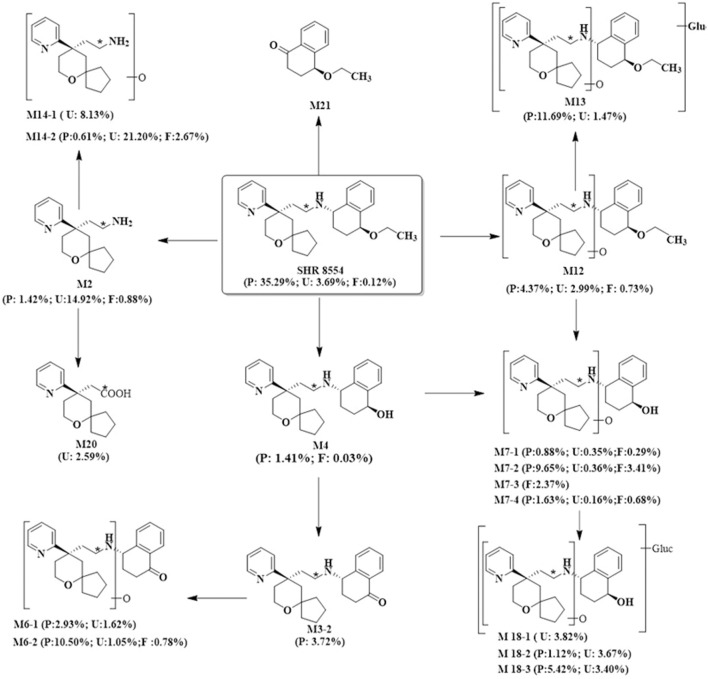
Possible metabolic pathways of SHR8554 in humans. Note: * indicates the position of 14C-labeling; P represents plasma, expressed as %AUC; U and F represent urine and faeces, expressed as %Dose.

According to the results of SHR8554 metabolite identification, we hypothesized that the main metabolic pathways of SHR8554 in male healthy subjects involve 1) N-dealkylation; 2) O-deethylation; 3) mono-oxidation; and 4) glucuronidation. The major biotransformation pathways of presumed SHR8554 in humans are shown in Figure 3.

## Discussion

SHR8554 is an injectable biased μ-opioid receptor agonist which exhibits selective activation of the G protein-coupled pathway but weaker recruitment of *ß*-arrestin-2. SHR8554 exerts good analgesic effects, although it also has weak side effects of gastrointestinal and respiratory depression. Our present study investigated the PK characteristics and major metabolic and clearance pathways of SHR8554 after a single ^14^C-labelled intravenous injection of dose mg/kg in healthy Chinese male subjects. Clinical studies of SHR8554 in humans have shown a good efficacy and safety profile.

The administered dose in the study was similar to that of the reference drug OLINVYK, which is a μ-opioid receptor (MOR)-biased agonist (generic name Oliceridine, TRV130) developed by Trevena that was approved for marketing in the US in 2020 (Azzam and Lambert, 2022). The radiation dosage assigned to the subjects was calculated to ensure safety. For a male subject weighing 60 kg, the entire body’s total dose was predicted as roughly 0.24 mSv (24 mrem), and there was minimal individual exposure risk in accordance with the standards of the International Commission on Radiological Protection (ICRP). A total of 33 subjects were screened, and six were finally recruited in this study. All six subjects had good medication compliance and completed the collection of sample and safety assessment following the protocol. [^14^C] SHR8554 was detected and quantified by radioactivity and mass spectrometry in the study.

After a single intravenous injection of [^14^C] SHR8554 (1 mg/41.3 μCi) in healthy male subjects, the overall recovery was 99.68% ± 1.56% within 216 h, generally achieving mass balance. Urine was the main route of excretion, accounting for 76.22% of the administered radioactivity, with only 3.69% in its original form. In addition, faecal elimination accounted for 23.46% of the administered radioactivity, with only 0.12% in its original form. Moreover, the excretion of total radioactivity in the urine and faeces occurred mainly within 72 h (3 days) after dosing, and the average excretion accounted for approximately 93.90% of the administered dose. As SHR8554 is mainly excreted in the urine in humans, the risk of PK alterations due to impaired renal function should be considered.

The PK results showed that after a single intravenous administration of [^14^C] SHR8554 injection in subjects, the peak time of total plasma radioactivity (isomolar amount of SHR8554) was 0.167 h for median T_max_, which was the same as that of the parent drug. The mean C_max_ of [^14^C] SHR8554 was 13.8 ± 3.94 ng Eq./g, while the C_max_ of the parent drug was 11.9 ± 3.81 ng/g. The mean terminal elimination half-life t_1/2_ was 10.4 ± 3.11 h. The PK parameters of SHR8554 in plasma were similar to those described in previous studies. The concentration of radioactivity decreased progressively after the peak was reached, and only a small amount of radioactivity could be detected in the plasma of two subjects 48 h after administration. The radioactivity was below the lower limit of quantification (0.438 ng Eq./g) in the four subjects, and the blood-to-plasma ratios of total radioactivity ranging from 0.370 to 0.813 at each time points, suggesting that there was no significant binding between [^14^C] SHR8554, including its metabolites and blood cells.

**FIGURE 4:**
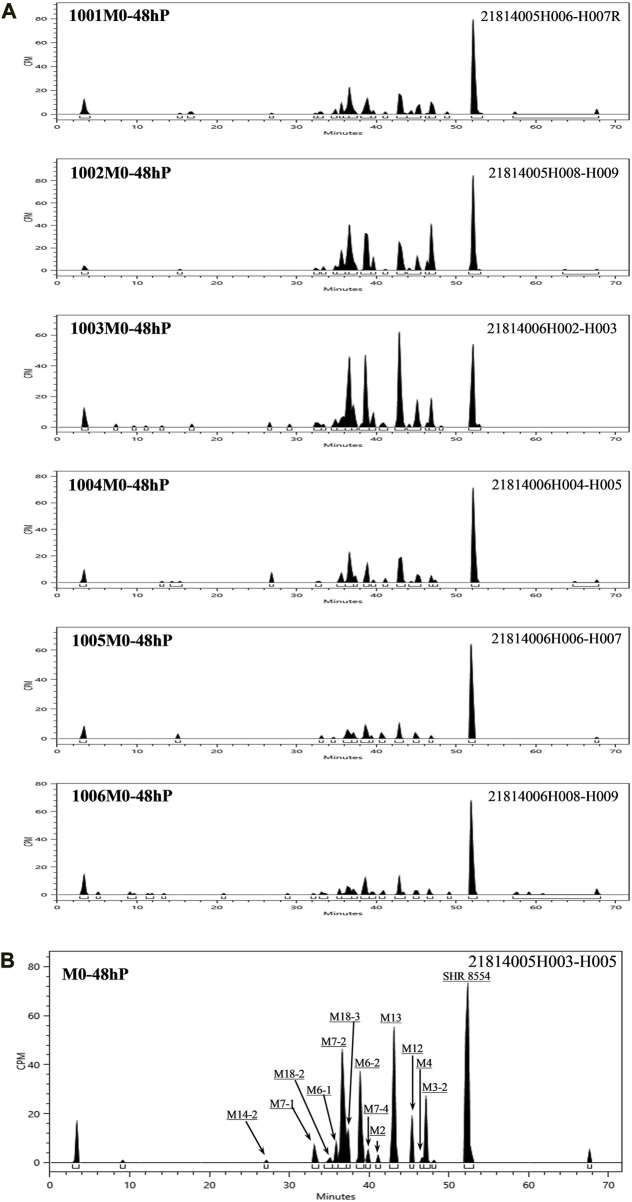
**(A)**: Radioactive liquid chromatography profiles of plasma samples from healthy male subjects 1001-1006 at 0 h–48 h. **(B)**: Radioactive liquid chromatography profile of mixed plasma samples from six healthy male subjects at 0 h–48 h.

**FIGURE 5:**
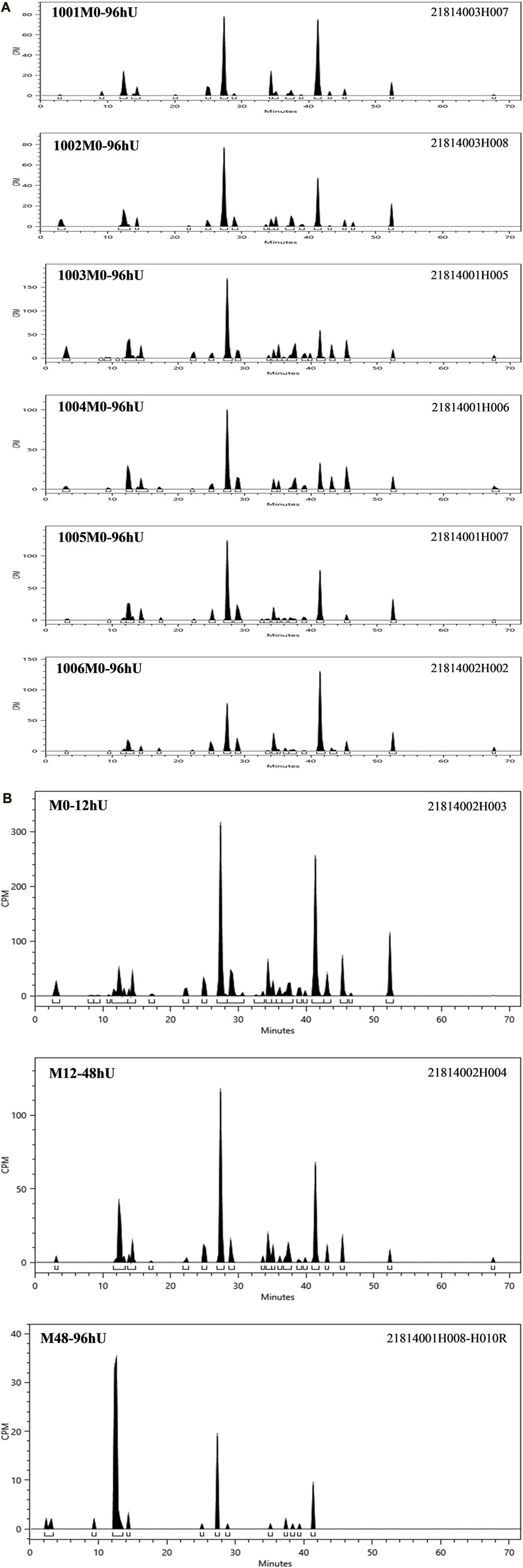
**(A)**: Radioactive liquid chromatography profile of urine samples from healthy male subjects 1001-1006 at 0 h–96 h. **(B)** Radioactive liquid chromatography profile of mixed urine samples from six healthy male subjects at 0 h–96 h.

**FIGURE 6:**
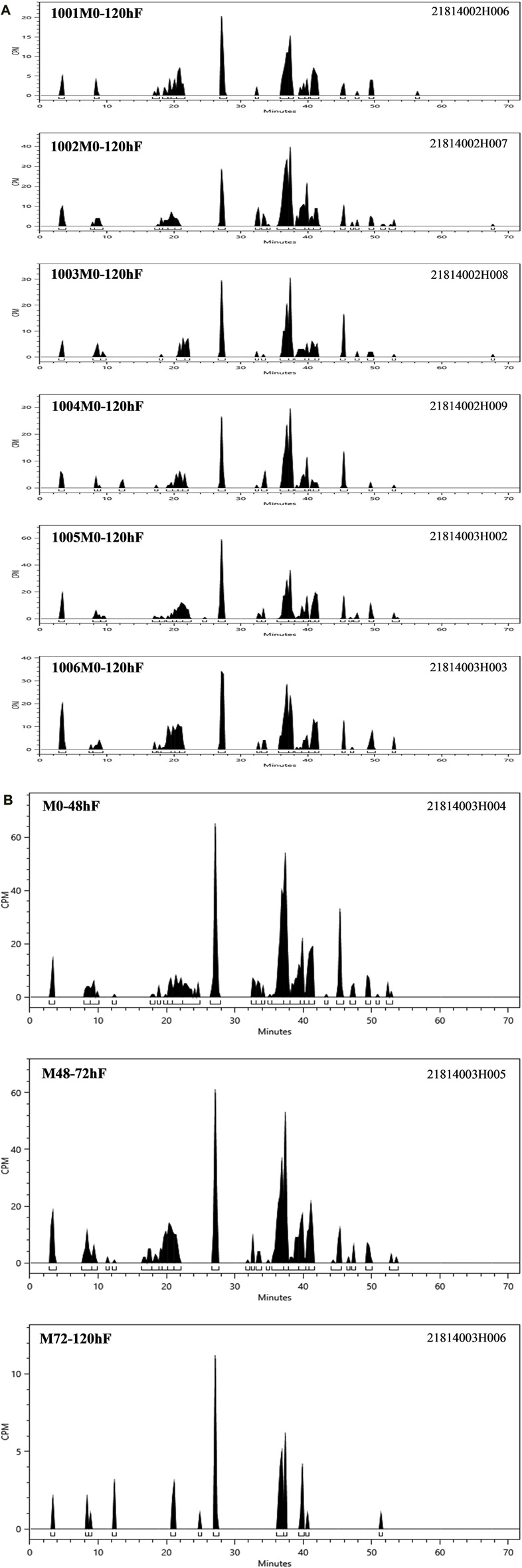
**(A)**: Radioactive liquid chromatography profile of faecal samples from healthy male subjects 1001-1006 at 0 h–120 h. **(B)** Radioactive liquid chromatography profile of mixed faecal samples from six healthy male subjects at 0 h–120 h.

According to the results of the metabolite study, it can be concluded that in addition to the parent drug, a total of 17 major metabolites were analysed and identified in blood, urine and faeces in this study. In plasma, the major metabolite in humans was M13 (11.69% of the total plasma radioactivity exposure). In urine, the major metabolite in humans was M14-2 (21.20% of the administered dose). In faeces, the main metabolite in humans was M7-2 (3.41% of the administered dose). The metabolic pathways involved 1) N-dealkylation; 2) O-deethylation; 3) mono-oxidation; 4) glucuronidation, etc. In addition to the parent drug, a total of 17 major radioactive metabolites were identified, including N-dealkylation metabolite (M2), O-deethyl and dehydrogenated metabolite (M3-2), O-deethyl metabolite (M4), mono-oxidation metabolites of M3 (M6-1 and M6-2), mono-oxidation metabolites of M4 (M7-1, M7-2, M7-3, and M7-4), mono-oxidation metabolite of the parent drug (M12), further glucuronidation metabolite (M13), mono-oxidation metabolites of M2 (M14-1 and M14-2), glucuronidation metabolites of M7 (M18-1, M18-2, and M18-3), and carboxylic acid metabolite formed through further oxidation of M2 (M20). Additionally, one metabolically related oxidized N-dealkylation metabolite (M21) was detected by mass spectrometry. The radioactive liquid chromatography profiles of plasma samples from six male healthy volunteers at 0 h–48 h are shown in Figures 4A, B. The radioactive liquid chromatography profiles of urine samples from 0 h to 96 h are shown in Figures 5A, B. The radioactive liquid chromatography profiles of faecal samples from 0 h to 120 h are shown in Figures 6A, B.

Preclinical studies have shown that SHR8554 activates G-protein with an efficacy comparable to that of morphine and OLINVYK, whereas the effect on *ß*-arrestin recruitment is approximately one-tenth that of morphine and OLINVYK (Sverrisdóttir et al., 2015). This study also demonstrated that the overall safety profile of [^14^C] SHR8554 was good with no unexpected safety risks. Except for one case of moderate TRAE (vomiting), all other TRAEs (nausea, dizziness, malaise) were mild, and no serious adverse events occurred.

In summary, we report the mass balance, metabolism, excretion and safety of SHR8554 in healthy Chinese subjects. The mean cumulative excreted radioactivity reached 99.6% in 216 h, which suggested that excretion of radioactivity was nearly complete in human. The main metabolic pathways of SHR8554 in humans involved N-dealkylation; O-deethylation; mono-oxidation; glucuronidation. The main excretion route of SHR8554 is urinary system. Moreover, SHR8554 showed safety profile at the dose used in this study.

## Data Availability

The raw data supporting the conclusion of this article will be made available by the authors, without undue reservation.
